# Target identification and validation of the alpha7 nicotinic acetylcholine receptor as a potential therapeutic target in retinal disease

**DOI:** 10.3389/fopht.2023.1190439

**Published:** 2023-07-24

**Authors:** David M. Linn

**Affiliations:** Department of Biomedical Sciences, Grand Valley State University, Allendale, MI, United States

**Keywords:** retina, nicotinic acetylcholine receptor (nAChR), α7 nAChR, retinal ganglion cell (RGC), neuroprotection, neurogenesis

## Abstract

The role of acetylcholine (ACh) in visual processing in the mammalian retina has been the focus of research for many decades. Pioneering work on the localization of ACh discovered that the neurotransmitter is synthesized and stored in a distinct subpopulation of amacrine (starburst) cells. It has been shown that ACh release is regulated to a low resting “tonic” level, much like what is observed at the neuromuscular junction (NMJ). If there were a dysfunction in the tonic release of ACh, might post-synaptic changes render the targets of ACh [i.e., retinal ganglion cells (RGCs)] vulnerable to disease? During my time at Pharmacia & Upjohn (PNU), selective nicotinic ACh receptor (nAChR) agonists (e.g., PNU-282987) were developed as a possible therapy for central nervous system (CNS) diseases. As RGCs are the main targets of neurodegeneration in glaucoma, could the activation of this target provide neuroprotection? In response to this question, experiments to identify alpha7 nAChRs in the retina (i.e., target ID studies) followed by “proof-of-concept” experiments were conducted. Target ID studies included binding studies with retinal homogenates, [^125^I]-alpha-bungarotoxin (α-BTX) autoradiography, and fluorescently tagged α-BTX binding in retinal slices. Imaging studies of intracellular calcium dynamics in the retinal slice were conducted. Reverse transcription-polymerase chain reaction (RT-PCR) analysis with alpha7 nAChR knockout mice using the “laser-capture microdissection” technique, *in situ* hybridization studies, and RT-PCR analysis of the human retina were conducted. Collectively, these experiments confirmed the presence of alpha7 nAChRs on specific cells in the retina. “Proof-of-concept” neuroprotection studies demonstrated that PNU-282987 provided significant protection for RGCs. This protection was dose dependent and was blocked with selective antagonists. More recently, evidence for the generation of new RGCs has been reported with PNU-282987 in rodents. Interestingly, the appearance of new RGCs is more pronounced with eye-drop application than with intravitreal injection. One could postulate that this reflects the neurogenic activation of alpha7 receptors on the retinal pigment epithelium (RPE) (eye drops) vs. a neuroprotective effect on RGCs (injections). In conclusion, there does appear to be a cholinergic retinal “tone” associated with RGCs that could be utilized as a neuroprotective therapy. However, a distinct cholinergic neurogenic mechanism also appears to exist in the outer retina that could possibly be exploited to generate new RGCs lost through various disease processes.

## Introduction

The cholinergic system of the mammalian retina has been investigated for many decades. It is widely accepted that the cells in the mammalian retina that synthesize and use acetylcholine (ACh) as a neurotransmitter are a well-defined population of amacrine cells comprising 3% of the entire amacrine cell population (1). These cells have a distinctive radial symmetry and are commonly referred to as the “starburst” amacrine cells (2–7). As the starbursts are the only neurons in the retina that synthesize ACh, the release of ^3^H-ACh (after loading with ^3^H-Ch) is taken to reflect the combined activity of the entire cholinergic population (8–10). The excitatory cone bipolar cell input is mediated *via* glutamate and several glutamate analogs evoke massive release (11), although the blocking of a specific subtype blocks responses to light-flash stimulation (12–14).

Starburst amacrine cells synthesize and release both γ-aminobutyric acid (GABA) and ACh when depolarized (8, 9, 15). The amount of ACh released from the rabbit retina increases at both light onset and light offset, and is maximally stimulated by large stimuli at temporal frequencies of 1–4 Hz (9, 16, 17). The majority of the synaptic output from the starburst cells is directed at retinal ganglion cell (RGC) dendrites (18–20). Starburst amacrine cells are known to provide a major synaptic input to directionally selective (D/S) RGCs, but it is uncertain what role they play in establishing the selectivity of their responses [reviewed in (21)]. In an elegant study (featured on the cover of *Nature*), He and Masland (22) photoablated labeled starbursts on the preferred side and null side of D/S RGCs and did not observe a significant change in directional selectivity. However, in another noteworthy study, Yoshida et al. (23) performed intravitreal injections of a toxin selective for starburst amacrines in a transgenic mouse model that resulted in the near elimination of all starbursts and with them all D/S responses from ganglion cells. The possible differences between these two results were reviewed by Wassle (24). Sethuramanujam et al. (25) have provided evidence for the concept of a mixed ACh/GABA transmission in the direction coding in the retina. In 2022, Kim et al. stated “there is a striking discontinuity in our current understanding of the neural coding of motion direction” (26). Subsequently, they [and others (27)] reported evidence for D/S starbursts in the primate retina. Previously, D/S was thought to occur at the cortical level in primates. In addition, a new layer of complexity has been proposed, with evidence suggesting a role in D/S processing at the bipolar cell level (28, 29). Overall, there does not appear to be universal agreement on the exact role played by ACh released from the starburst amacrine cells in D/S among different mammals.

Does ACh play a role in retinal physiology other than directional selectivity that needs to be considered? Other than the rapid depolarizations observed with nicotinic ACh receptor activation in other parts of the nervous system, what else is associated with ACh release? Massey and Redburn (17) reported that ACh release from the rabbit retina was “tonically” inhibited by GABA to a relatively low resting level. This level of release could be greatly enhanced by selective GABA inhibitors (30). One could postulate that this can be compared with the tonic release of ACh at the neuromuscular junction (NMJ). The tonic release at the NMJ is essential for maintaining the functionality of that synapse. It is well known that regular exercise increases muscle tone. Could a similar mechanism be at work at the starburst-to-RGC synapse in the retina? Could there be a “cholinergic tone” mechanism at that synapse that is essential for proper functioning? Importantly, could the disruption of this mechanism be involved in certain retinal diseases? Using the hypertonic saline model of ocular hypertension in rats, Cooley-Themm et al. (31) found that starburst amacrine cell numbers, ACh content, and α7 nicotinic ACh receptor (nAChR) protein expression all began to decrease 1 week after the procedure to induce glaucoma-like conditions. This preceded the significant loss of RGCs that typically occurs 1 month after the same procedure. Therefore, as it remains highly speculative, the temporal sequence of the disruption of cholinergic transmission (i.e., loss of “cholinergic retinal tone”) and the loss of RGCs seen in glaucoma needs further experimental support.

Although the primary risk factor for glaucoma is considered to be elevated intraocular pressure (ocular hypertension), excitotoxicity is another possible mechanism. Excitotoxicity (neuronal cell death caused by excessive excitatory input) has long been linked to various diseases of the central nervous system (32, 33), including the retina. In the retina, diseases associated with excitotoxicity include retinal ischemia, diabetic retinopathy, and glaucoma (34, 35). Several studies have identified an excess of the excitatory neurotransmitter, glutamate, in the vitreous humor (36–38). Landmark studies have demonstrated that excess glutamate release in the eye leads to a prolonged influx of non-specific cations in RGCs and triggers intracellular signaling cascades, leading to apoptosis (39, 40). Interestingly, exposure to cigarette smoke reduces kainic acid-induced neurotoxicity in rats (41). In addition, the stimulation of nAChRs has been reported to inhibit β-amyloid toxicity (42, 43). Nicotine has also been reported to protect against *N*-methyl-D-aspartate (NMDA)- and glutamate-induced neurotoxicity (44), with Kaneko et al. (45) proposing that the α7 nAChR plays a role in the observed protection. Researchers at Abbott Laboratories reported that ABT-418 and nicotine can protect against glutamate-induced toxicity in rat cortical cell culture experiments (46). In neuroprotection studies utilizing adult porcine RGCs isolated by a “panning” procedure, Wehrwein et al. (47) demonstrated that ACh, nicotine, and choline significantly reduced glutamate-induced excitotoxicity through α-bungarotoxin-sensitive nAChRs. Choline is regarded as a relatively selective agonist and α-bungarotoxin (α-BTX) as a relatively selective antagonist for the α7 nAChR.

Are there highly selective α7 nAChR agonists available (48, 49) to further investigate the role of ACh in the “cholinergic tone” model of glaucoma? The proposed sequence being that if starbursts are lost during the development of elevated intraocular pressure (IOP), then the release of ACh should decrease. One could reason that activation of the α7 nAChR on RGCs (if they exist) with a selective agonist could restore “tone”. This would be comparable to the approach of blocking acetylcholinesterase (AChE) with selective inhibitors (to increase ACh levels) to counteract the loss of cholinergic neurons in Alzheimer’s disease, or selectively activating dopamine receptors with agonists to counter the loss of dopaminergic neurons in Parkinson’s disease. PNU-282987 was developed at Pharmacia & Upjohn in the early 2000s. Binding studies in rat chimera cells using PNU-282987 demonstrated that it is a potent and specific agonist for the α7 nAChR (50, 51). These studies demonstrated that methyllycaconitine (MLA), a specific α7 nAChR antagonist, competitively bound to α7 nAChRs when both PNU-282987 and MLA were present in brain tissue. In electrophysiology studies using rat hippocampal neurons, PNU-282987 evoked a rapidly desensitizing inward whole-cell current associated with the opening of the α7 nAChR channel. This current was eliminated if MLA was introduced before PNU-282987 (50). Overall, there is compelling evidence that PNU-282987 can be regarded as a highly selective α7 nAChR agonist. However, before testing PNU-282987 as a potential therapy in the retina, one needs to demonstrate that there are α7 nAChRs present in the mammalian retina (target identification) and then demonstrate that PNU-282987 is effective in appropriate model systems (validation). Preliminary experiments exploring target identification and validation of the α7 nAChR (Chrna7) in the retina will be presented in this report with results from binding studies, the imaging of intracellular calcium dynamics, reverse transcription-polymerase chain reaction (RT-PCR) studies, *in situ* hybridization (ISH), and isolated mammalian RGC culture experiments.

## Methods

### Binding assays

Porcine eyes were obtained from a local meat processing company (Pease Packing Co., Scotts, MI, USA) and kept on ice. Retinas were removed and homogenized using a rotating pestle. The homogenate was centrifuged at 1,000 ×g for 10 minutes at 40°C. The supernatant was collected and centrifuged at 20,000 ×g for 20 minutes at 40°C. The resulting pellet was resuspended to a protein concentration of 1–8 mg/mL. Aliquots of 1 mL of homogenate were frozen at –80°C until needed for the assay. On the day of the assay, aliquots were thawed at room temperature and diluted with Kreb’s 20 mM HEPES [4-(2-hydroxyethyl)-1-piperazineethanesulfonic acid] buffer (Sigma-Aldrich, St. Louis, MO, USA) (pH 7.0) and 25–150 μg of protein was added per test tube. Protein concentrations were determined by the Bradford method (52) using bovine serum albumin (BSA) (Sigma-Aldrich) as the standard.

In the [^125^I]-α-BTX binding assay, 0.4 mL of homogenate was added to test tubes containing buffer plus 0.1% BSA and 10 μM PMSF/test binding compound and radioligand ([^125^I]-α-BTX). Homogenates were incubated in a final volume of 0.5 mL for 4 hours at 37°C. Non-specific binding was determined by 1 nM α-BTX (Sigma-Aldrich). In line with the methods for competition studies, the drugs were added in increasing concentrations to the test tubes before the addition of [^125^I]-α-BTX (2,000 Ci/mmol; New England Nuclear, Boston, MA, USA), up to a final concentration of 0.1 nM. The incubations were terminated by rapid vacuum filtration through Whatman GF/B glass filter paper mounted on a 48-well Brandel cell harvester. Filters were pre-soaked in 50 mM Tris-HCl (pH 7.0) and 0.05% polyethylenimine. The filters were rapidly washed two times with 5-mL aliquots of cold 0.9% saline and then measured for radioactivity by liquid scintillation spectrometry.

[^3^H]-cytisine (35 Ci/mmol; New England Nuclear) was used to label the α4 nAChR and [^3^H]-epibatidine (48 Ci/mmol; New England Nuclear) in order to label the α3 nAChR in porcine retinal membranes. The final radioligand concentration used was 1.0 nM. In the assays, 0.4 mL of homogenate was added to test tubes containing buffer/test compound and radioligand, and were incubated in a final volume of 0.5 mL for 1 hour. Non-specific binding was defined by 1 mM nicotine (Sigma-Aldrich). For the α3 binding assay, 30 nM of cold cystine was used to block the endogenous α4 nAChR. The incubations were terminated by rapid vacuum filtration through Whatman GF/B glass filter paper mounted on a 48-well Brandel cell harvester. Filters were pre-soaked in 50 mM Tris-HCl (pH 7.0) and 0.05% polyethylenimine. The filters were rapidly washed two times with 5-mL aliquots of cold 0.9% saline and then measured for radioactivity by liquid scintillation spectrometry.

The inhibition constant (Ki) was calculated from the concentration-dependent inhibition of radioligand binding obtained from a non-linear regression-fitting program according to the Cheng-Prusoff equation (53). Hill coefficients were obtained using non-linear regression (GraphPad Prism sigmoidal dose–response with a variable slope). The Ki is presented as the mean ± standard error of the mean (SEM) for each inhibitor of α-BTX (*n *= 3), for cytisine (*n* = 3), and for epibatidine (*n* = 3). Percent inhibition is presented as the mean ± SEM for each inhibitor of α-BTX (*n*=4), for cytisine (*n* = 3), and for epibatidine (*n* = 3) at set doses.

### Autoradiography

Before incubation with [^125^I]-α -BTX, a series of coronal eye sections from each rat was incubated in binding buffer at 22°C for 10 minutes. After the preincubation step, the samples were incubated with 2 nM [^125^I]-α -BTX for 4 hours at 22°C. An adjacent series of sections from each rat was used to determine non-specific [^125^I]-α-BTX binding (in the presence of 1 mM nicotine bitartrate). The slides were then washed as follows: 10 minutes in binding buffer (twice), 5 seconds in 0.1 × binding buffer (twice), 5 seconds in 5 mM HEPES (pH 7.5) (twice), and finally with dH_2_O twice. The binding buffer (pH 7.5) comprised the following: 144 mM NaCl, 1.5 mM KCl, 2 mM CaCl_2_, 1 mM MgSO_4_, 200 mM Tris-HCl, 20 mM HEPES, and 0.1% BSA (w/v; weight to volume). All chemicals were obtained from Sigma-Aldrich.

### Toxin binding studies in the retinal slice

Retinal slices were prepared as below. Instead of loading cells with fluo-4 AM, Texas Red-tagged α-BTX (Sigma-Aldrich) was added to dishes at a concentration of 100 nM for 30 minutes, followed by a 30-minute wash prior to imaging on a deconvoluting microscope.

### Calcium dynamics in retinal slices

Rat retinal slices were prepared according to procedures adapted from previous methods (54). Each eye was excised and hemisected, and the anterior chamber was discarded. Two radial incisions were made with fine scissors such that the eyecup would lay flat with dorsal and ventral hemispheres. A black Millipore filter (HABP 045; Merck Millipore, Burlington, MA, USA) was placed on top of the remaining vitreous and the retina flipped over on top of a Whatman #1 filter (Sigma-Aldrich, St. Louis, MO, USA). The Whatman was allowed to draw the vitreous through the Millipore to facilitate attachment of the retina to the Millipore. The retina/filter was then placed under a dissecting scope and the sclera peeled away, leaving the retina attached to the filter paper. The retina was then sliced with a fresh razor blade on a conventional razor-blade tissue slicer (Stoelting, Chicago, IL, USA). Slice thickness was 150 μm and controlled with the micrometer drive on the slicer. Individual slices were transferred to a 35-mm culture dish (three slices/dish) containing Ames buffer (Sigma-Aldrich), rotated such that the slices lay on their side with their ends anchored in Vaseline® tracks.

### Fluorescence measurements

For the measurement of calcium dynamics (55, 56) in retinal slices, Ames buffer was replaced with a loading solution [one part Ames to two parts phosphate-buffered saline (PBS) without Ca^++^]. The loading solution contained the fluorescent calcium indicator fluo-4 AM [10 μL of a 1 mM solution of dimethylsulfoxide (DMSO) into a 15 mL loading solution; Molecular Probes, Thermo Fisher Scientific, Waltham, MA, USA]. After a 1-hour loading period in a tissue-culture incubator, the loading solution was replaced with fresh Ames for a recovery period (i.e., “wash phase”) of 30 minutes at 37°C. For binding experiments, Texas Red-tagged α-BTX was added to dishes at a concentration of 100 nM for 30 minutes followed by a 30-minute wash.

Dishes were transferred to an inverted microscope (Nikon) equipped with a perfusion system (Warner Instruments, Holliston, MA). Fluorescence within individual slices was examined with a deconvoluting microscope (MetaMorph Imaging Systems, Molecular Devices, San Jose, CA, USA) until a suitable region of a slice was chosen for examination. Regions of relatively uniform focal plane and labeling were determined as “suitable regions” for analysis. Areas to be monitored were designated by drawing polygons around labeled cells in the ganglion cell layer. Images (60×; 1.0 NA) were acquired in 1-μm steps at each time point with a frame duration of 200 milliseconds and stacks acquired at 10-second intervals. There was no correction for bleaching. Drugs were applied in 1-minute bath-applied pulses, and a 10-minute wash-out between doses was followed. Digitized images were captured in the form of stacks and calcium dynamics in the designated areas of the slice were analyzed using the MetaMorph analysis software. Data in the form of spreadsheets were exported to Excel (Microsoft) and then SigmaPlot (Jandel Scientific) for the generation of “brightness-over-time” graphs. Dose–response data were obtained by measuring the peak response, divided by the basal response immediately prior to drug application. Dose–response experiments were considered completed when the maximal change in fluorescence was taken as the saturating dose. The change delta (Δ) in fluo-4 fluorescence (fluorescence-max/fluorescence-initial) was plotted against concentration. Dose–response curves were generated using a “best fit” program in Excel. Nicotine and epibatidine were obtained from commercial sources (Sigma-Aldrich, St. Louis, MO), whereas AR-R-17779 was obtained from “in-house”. Values from each concentration tested are presented as mean ± SEM *(n* = 4).

### RT-PCR of retinal tissue

Cryosections of eyes from wild-type and α7 knockout mice were run through a dehydration series in preparation for laser-capture microdissection (LCM; Arcturus, Thermo Fisher Scientific, Waltham, MA). In brief, the inner portion of each retinal section [corresponding to the retinal ganglion cell layer (GCL)] was removed from the wild-type and knockout mice. This consisted of clumps of cells that could include RGCs and displaced amacrine cells. Total RNA was extracted from the laser-captured material and put through one round of linear amplification prior to use in PCR, which followed standard cycling parameters. Mouse hippocampal cDNA served as a positive control, whereas dH_2_O served as a negative control. Subsequent sequencing from both ends of the PCR product confirmed α7 transcript.

Human ocular tissue was obtained from the Michigan Eye Bank. Total RNA was prepared, and reverse transcription carried out to generate retinal cDNA. The primers were designed with the forward primer in exon 2 and the reverse primer in exon 6 such that only amplification from cDNA, as opposed to contaminated genomic DNA, would produce the correct size band of 450 bp (amplification from genomic DNA would create a much larger product). The α7 nAChR PCR reaction products from human retina cDNAs were generated using the human α7 nAChR-specific primer set. Human hippocampal cDNA was used as a positive control with SHEP-1 cells and sterile dH20 as negative controls for the PCR reactions. In addition, primers for β-actin were used on all of the cDNA samples to demonstrate that the cDNA was intact and of sufficient quality to use in PCR. Corresponding bands with the proper molecular weight (MW) were only detected in hippocampal and retinal cDNA and not in SHEP-1 cDNA and dH2O. Sequencing of the 450-bp PCR product made from human retinal cDNA confirmed that it matched exactly the functional human α7 cDNA sequence (GenBank® accession U62436).

### *In situ* hybridization

For tissue preparation, rat brain and eyes were rapidly obtained *post mortem* and embedded fresh in OCT and flash frozen in isopentane cooled in liquid nitrogen. Blocks were stored at –80°C. Sections of approximately 8 μm were obtained using the CryoJane Tape Transfer System (Leica Biosystems, Deer Park, IL) and were used immediately for ISH.

For probe preparation, rat α7 nicotinic acetylcholine receptor (α7 nAChR) cDNA was cloned into pBluescript SK(+) at the *Eco*R1 site of the multiple cloning sequence. The plasmid was linearized with *Kpn*1 and *Sma*1 to serve as the template for an antisense and sense probe, respectively. Full-length (1,508 kb) rat α7 nAChR antisense and sense digoxigenin (DIG)-labeled transcripts were generated using the DIG RNA Labeling Kit (Roche, Basel, Switzerland). T3 ribonuclease was used to generate the antisense probe and T7 was used to generate the sense probe. Fidelity of transcription was assessed using gel electrophoresis. Approximate yield was determined using DIG Quantification and Control Test Strips (Roche). An approximately 600-bp antisense probe for mouse β-actin was used as a positive control.

For the ISH procedure, the non-isotopic ISH method used was a modification of the procedures of Braissant et al. (57) and Yang et al. (58). Basically, following several prehybridization steps, tissues were hybridized overnight then, after a series of posthybridization steps and stringency washes, the slides were prepared for immunodetection using a tyramide signal amplification protocol. Immunodetection and amplification were performed on a Dako Autostainer (Agilent, Santa Clara, CA, USA).

The experimental matrix comprised two tissues: (1) rat brain tissue (positive control); and (2) rat eye tissue.

Four probes were used in this experiment:

1) a7 nAChR antisense;
2) a7 nAChR sense;
3) β-actin antisense (technique positive control); and
4) neomycin antisense (an antisense probe against non-coding mRNA).

### Cell culture/neuroprotection studies

Retinas were removed from fresh porcine eyes donated by DeVries Meats, Inc., Coopersville, MI, USA. Papain was added to enzymatically dissociate the cells, and trituration was utilized for further dissociation. The individual retinal cells were suspended in Dulbecco’s modified Eagle’s medium (DMEM) CO_2_-independent medium (Gibco, Carlsbad, CA, USA). A two-step panning procedure modified from Barres et al. (59) was used to isolate the retinal ganglion cells. In the first step, the retinal cell suspension was applied to dishes coated with IgG goat anti-rat antibody. The IgG antibody captured cells with low affinity for RGCs. After incubation at 36°C, the cell suspension was transferred to plates coated with IgM goat anti-mouse antibody tagged with the Thy 1.1 antibody. The Thy 1.1 maintained high affinity for RGCs. The plates were divided into three categories: control plates received no treatment; experimental plates received varying concentrations of agonist, either PNU-282987 or nicotine (Sigma-Aldrich), followed later by an application of glutamate; and other plates received glutamate only. The cells were allowed to incubate at 36°C for 3 days. Calcein (Sigma-Aldrich) was then applied to all plates, allowing the live cells to fluoresce when viewed at 495 nm under a microscope. Images were captured and a software program was utilized to count cells. Cell survival on the treatment plates was calculated as a fraction of control plate survival. Each experiment was carried out six times to determine pharmacological results. Statistical analysis was performed on data using an analysis of variance (ANOVA) followed by linear contrast. A *p*-value < 0.05 was considered statistically significant. Statistical treatments were performed on data normalized to control values for each experimental series to minimize variation.

## Results

**Figure 1:**
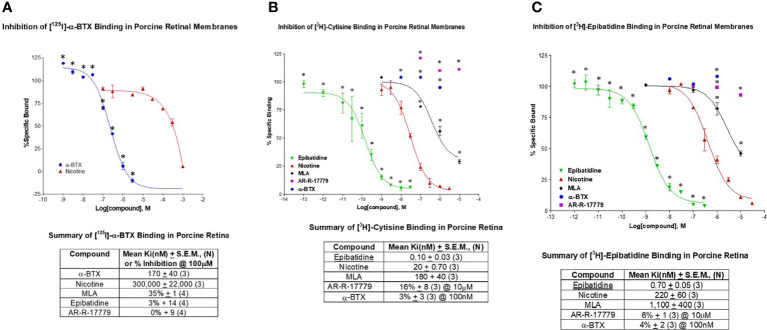
This figure displays the results of binding studies conducted with pig retinal homogenates. **(A)** The observed inhibition of [^125^I]-α-bungarotoxin (α-BTX) was consistent with the binding profile of α7 nAChRs. The mean Ki (nM) ± SEM was determined against α-BTX (*n* = 3) and nicotine (*n* = 3). Percent inhibition of binding (curves not shown) was determined against MLA (methyllycaconitine) (*n* = 4), epibatidine (*n* = 4), and AR-R-17779 (*n* = 4). **(B)** The inhibition of [^3^H]-cytisine (35 Ci/mmol) was consistent with the presence of the α4 nAChR. The mean Ki (nM) ± SEM was determined against epibatidine (*n* = 3), nicotine (*n* = 3), and MLA (*n* = 3). Percent inhibition of binding against AR-R-17779 (at 10 mM) and α-BTX was also determined. **(C)** The inhibition of [^3^H]-epibatidine (48 Ci/mmol) was taken to indicate the α3 nAChR. The mean Ki (nM) ± SEM for epibatidine (*n* = 3), nicotine (*n* = 3), and MLA (*n* = 3) were determined. Percent inhibition of binding against 10 mM AR-R-17779 (*n* = 3) and 100 nM α-BTX (*n* = 3) were also determined and indicated minimal activity at α7 nAChRs. Collectively, these results indicate the possible presence of different nAChRs in the porcine retina, including the α7 nAChR.

Binding studies were conducted on retinal homogenates to test for the presence of different nicotinic AChR subtypes. α-Bungarotoxin (α-BTX) is a neurotoxin that blocks neuromuscular transmission *via* irreversible inhibition of nAChRs at the pore opening (60). It is considered selective for α7 nAChRs over other nAChRs (61), including α3β4 receptors (IC_50_ values of 1.6 nM and > 3 μM, α7 and α3β4 respectively; Tocris Bioscience, Minneapolis, MN, USA). In **Figure 1**, binding against [^125^I]-α-BTX was consistent with the presence of α-BTX sensitive receptors in porcine retinal membranes. The mean Ki for α-BTX indicated high potency at 170 ± 40 nM, whereas nicotine had a mean Ki of 300 ± 22 μM (*n* = 3 for each). This is consistent with the relatively low affinity of nicotine compared with α-BTX for nAChRs. Methyllycaconitine (MLA) was the first low-molecular-weight ligand to be shown to discriminate between muscle nicotinic receptors and their α-BTX-binding counterpart in the brain (62). The percent inhibition by 100 μM MLA was determined to be 35% ± 1% (*n* = 4) for α-BTX, 3% ± 14% for 100 μM epibatidine, and 0% ± 9% for AR-R-17779. This is consistent with the way in which MLA interacts with the α7 pore at a distinct site, resulting in a lower affinity relative to α-BTX. Epibatidine is expected to display minimal affinity at α-BTX sites (high affinity expected at α3 nAChRs). A negligible interaction with the selective α7 agonist AR-R-17779 indicates a distinct, separate binding interaction with the agonist binding site on the α7 nAChR.

Cytisine is a partial agonist with high-affinity binding to the α4β2 nAChR. The nAChR is believed to be central to the rewarding effects of nicotine. It has been licensed as an aid for smoking cessation in Eastern Europe for 40 years and marketed as Tabex®. The mean Ki of epibatidine displayed high-affinity binding against [^3^H]-cytisine at 0.10 ± 0.03 nM, and the mean Ki of nicotine and MLA was 20 ± 0.70 nM and 180 ± 40 nM, respectively (*n *= 3 for each). The relatively low-potency Ki for MLA is suggestive of a nAChR other than the α7 nAChR. Percent inhibition of binding was 16% ± 8% (at 10 μM) for the selective α7 agonist AR-R-17779 and 3% ± 3% (at 100 nM) for α-BTX (*n* = 3 for each). The low level of percent inhibition of an α7 selective agonist and antagonist is indicative of minimal interaction with cytisine at α7 nAChRs. Subsequent published studies from our laboratory have supported the presence of α4 nAChRs on small porcine RGCs and α7 nAChRs on large porcine RGCs (63). Interestingly, Elgueta et al. (64) reported that cytisine was selective for the β4 subunit of nAChRs on retinal amacrine cells.

As stated above, epibatidine was originally considered highly potent but relatively non-selective for α4β2 and α3β4 nAChRs, with low affinity for the α7 AChR. The inhibition of [3^H^]- epibatidine by non-labeled epibatidine displayed a mean Ki of 0.70 ± 0.05 nM (*n *= 3), whereas nicotine displayed a mean Ki of 220 ± 0.60 nM (*n *= 3). These values are indicative of the high-potency agonist action of epibatidine at nAChRs compared with nicotine. However, the high Ki value for MLA with the low percent inhibition by AR-R-17779 and α-BTX is evidence of the low binding affinity of epibatidine at the α7 nAChR, but suggests the presence of other nAChR subtypes. Tocris (www.tocris.com) lists epibatidine as a high-affinity nicotinic agonist (Ki values = 0.02 and 233 nM for α4β2 and α7 nicotinic receptors respectively). Together, these results and others indicate the presence of different types of nAChRs [α3, (65); α4, (63, 66)] in the mammalian retina, including the α7 nAChR (67).

**Figure 2:**
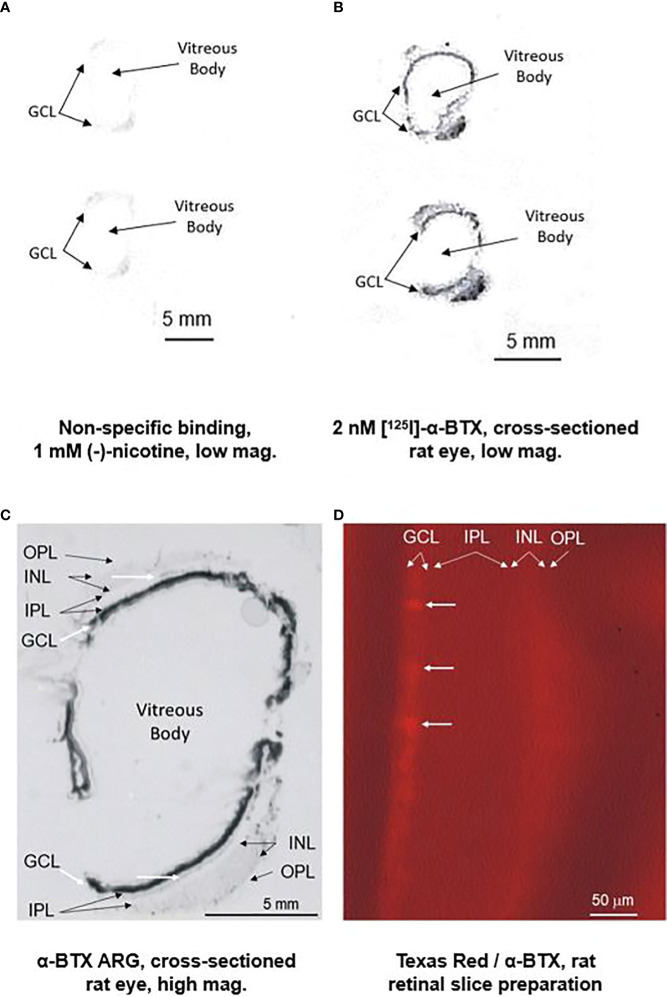
Preliminary ARG studies with [^125^I]-alpha-bungarotoxin and tagged alpha-bungarotoxin suggest localization of the α7 nAChR predominantly in the RGC layer (RGL). The top left figure **(A)** displays a low-power image of non-specific binding with 1 mM (-)-nicotine with a sectioned rat eye. The top right figure **(B)** displays a low-power image of binding with 2 nM [^125^I]-α-BTX. The figure on the lower left **(C)** is a close-up image showing heavy staining localized to the RGL of the rat eye, as indicated by the thick diagonal white arrows. Diffuse labeling is observed at the margin of the inner nuclear layer (INL) and the inner plexiform layer (IPL), as indicated by the thin horizontal white arrows. Although inconclusive at this magnification, this could coincide with the conventional location of the cholinergic “starburst” amacrine cells. Interestingly, there is diffuse labeling throughout the INL, consistent with reports of α7 nAChRs found on specific types of bipolar cells (28, 68). The figure on the right **(D)** is a higher-power magnification of Texas Red-tagged α-BTX labeling in a rat retinal slice preparation. Individual cells (horizontal white arrows) cannot be conclusively distinguished in the RGL.

Additional studies with α-BTX are shown in **Figure 2**. Part (A) shows a low-power image of autoradiography (ARG) of non-specific binding with nicotine. Part (B) shows low-power images of 2 nM [^125^I]-α-BTX ARG of an entire cross-sectioned rat eye. Part (C) is a higher-power image of a cross-sectioned rat eye exposed to [^125^I]-α-BTX. After processing, there is heavy staining localized to the ganglion cell layer (GCL) of the rat eye, as indicated by the diagonal white arrows. There is more diffuse labeling at the margin of the inner nuclear layer (INL) and the inner plexiform layer (IPL) indicated the horizontal white arrows. This could coincide with the location of the conventionally placed cholinergic “starburst” amacrine cells. However, the low-power magnification does not allow for the distinction of specific cells in the GCL or INL. Interestingly, there is diffuse labeling throughout the INL, which is consistent with reports of α7 nAChRs found on specific types of bipolar cells (28, 68). Part (D) is a higher-power magnification of Texas Red-tagged α-BTX labeling in a rat retinal slice preparation. Putative individual cells can be distinguished in the GCL. Recently, single-cell RNA sequencing (scRNA-seq) has emerged as a powerful tool to study cell-type-specific gene expression in the retina. As reviewed in (69), upward of 12 retina-specific scRNA-seq databases have been published on the NIH Gene Expression Omnibus (GEO). These publicly available databases can allow for cell-specific gene expression queries, including Chrna7 expression profiles.

**Figure 3:**
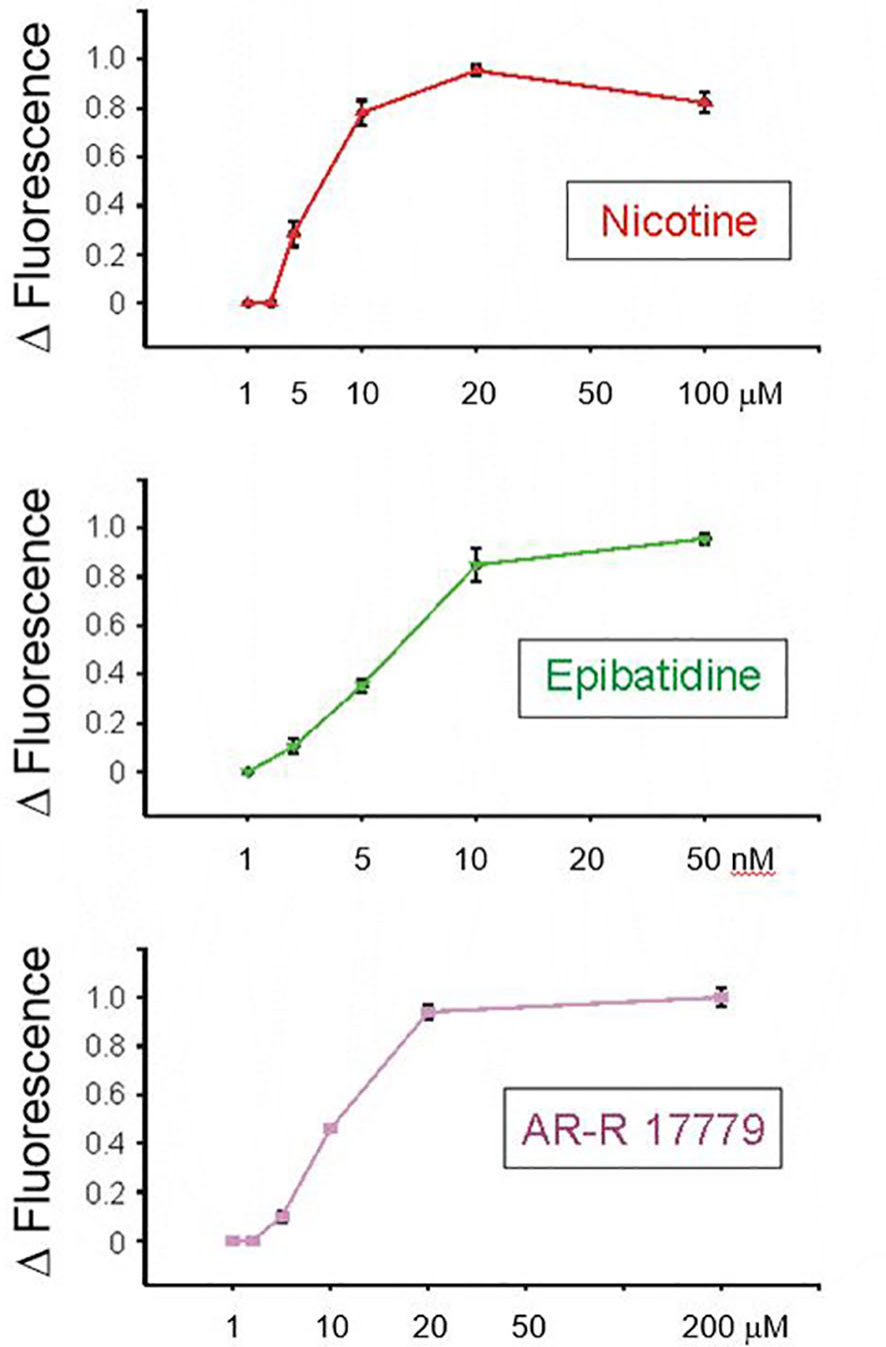
The intracellular calcium dynamics of individual retinal ganglion cells were examined in the rat retinal slice preparation. Results for nicotine, the non-selective, low-potency agonist for nicotinic receptors, are displayed at the top. Nicotine was observed to reach a threshold dose at 2 μM and a saturating dose at 20 μM. This is consistent with the presence of nicotinic AChRs on RGCs in the RGC layer. Epibatidine is a high-potency, but relatively non-selective, agonist at α3 receptors. As expected, the threshold for epibatidine was in the low nanomolar range (i.e., 1 nM) with a saturating dose between 10 and 20 nM. AR-R-17779 has been reported as a relatively high-affinity agonist with high selectivity for alpha7 nAChRs. AR-R-17779 reached a threshold dose at 2 μM and a saturating dose at approximately 20 nM.

The intracellular calcium dynamics of individual retinal ganglion cells were examined in the rat retinal slice preparation (*n* = 4 for each data point, mean ± SEM). The results for nicotine, the non-selective, low-potency agonist for nicotinic receptors, are displayed at the top of **Figure 3**. Nicotine was observed to reach a threshold dose at 2 μM and a saturating dose at 20 μM. This is consistent with cell culture experiments by Wehrwein et al. (47) who reported significant neuroprotection against glutamate toxicity from the range of 1 to 20 μM, with toxicity reported at 50 μM. This is consistent with the presence of nicotinic AChRs on RGCs in the RGL. Epibatidine is a high-potency, but relatively non-selective, agonist at α3 receptors. As expected, the threshold for epibatidine was in the low nanomolar range (1 nM), with a saturating dose between 10 and 20 nM. AR-R-17779 has been reported as a relatively high-affinity agonist with high selectivity for α7 nAChRs. AR-R-17779 reached a threshold dose at 2 μM and a saturating dose at approximately 20 μM. Collectively, these results and others support the existence of nAChRs on rat retinal ganglions as well as the existence of specific subtypes, such as the α7 nAChR.

**Figure 4:**
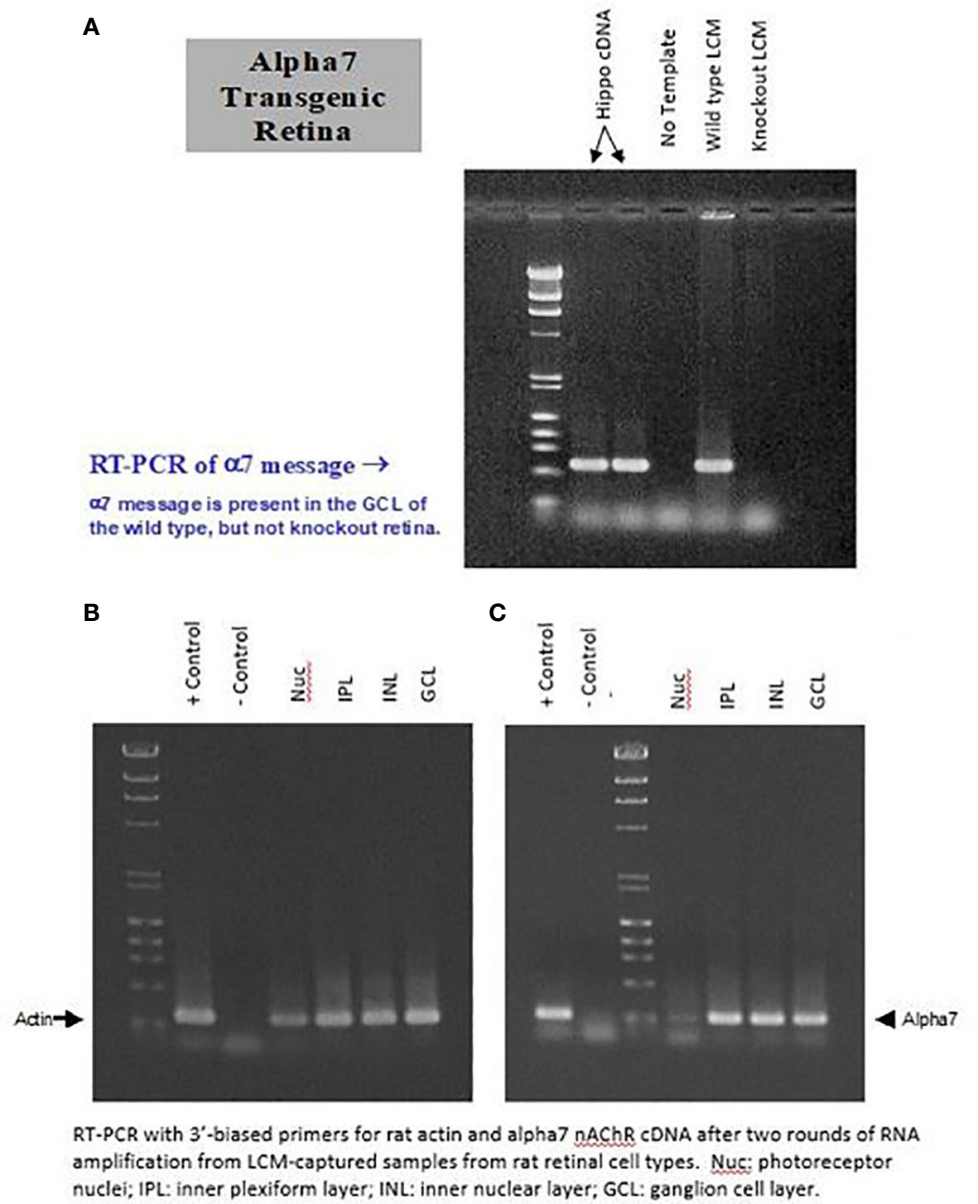
Clusters of cells from the GCL (RGCs and displaced ACs) were dissected from the retina using the “laser capture microdissection” (LCM) technique. LCM was conducted on the RGL from wild-type retina and from α7 knockout retina **(A)**. Two samples of hippocampal cDNA were used as a positive control for α7 nAChRs, whereas no template was used as a negative control. As shown in **(A)**, message was detected in hippocampal and wild-type tissue, whereas none was detected with the negative control and knockout retina. **(B)** shows the actin control. **(C)**, shows that mRNA was found in LCM cells from the GCL, INL, and IPL. A weak but clear band is present, corresponding to the photoreceptor nuclear (Nuc) layer.

RT-PCR experiments were conducted to determine if the mRNA for the α7 nAChR is present in the GCL of the rat retina. Cells from the GCL were dissected as clumps from retinal tissue using the LCM technique. LCM was conducted on the GCL from wild-type retina and from α7 knockout retina. Two samples of hippocampal cDNA were used as a positive control for α7 nAChRs, whereas no template was used as a negative control. As shown in the top of **Figure 4**, message was detected in hippocampal and wild-type tissue, whereas none was detected in the negative control and knockout retina. The bottom of **Figure 4** shows that mRNA was found in LCM cells from the GCL, INL, and IPL. There is a clear but weak band present in the photoreceptor nuclear (Nuc) layer. This confirms studies from other investigators (68, 70), who have localized α7 AChRs to bipolar cells, in addition to RGCs.

**Figure 5:**
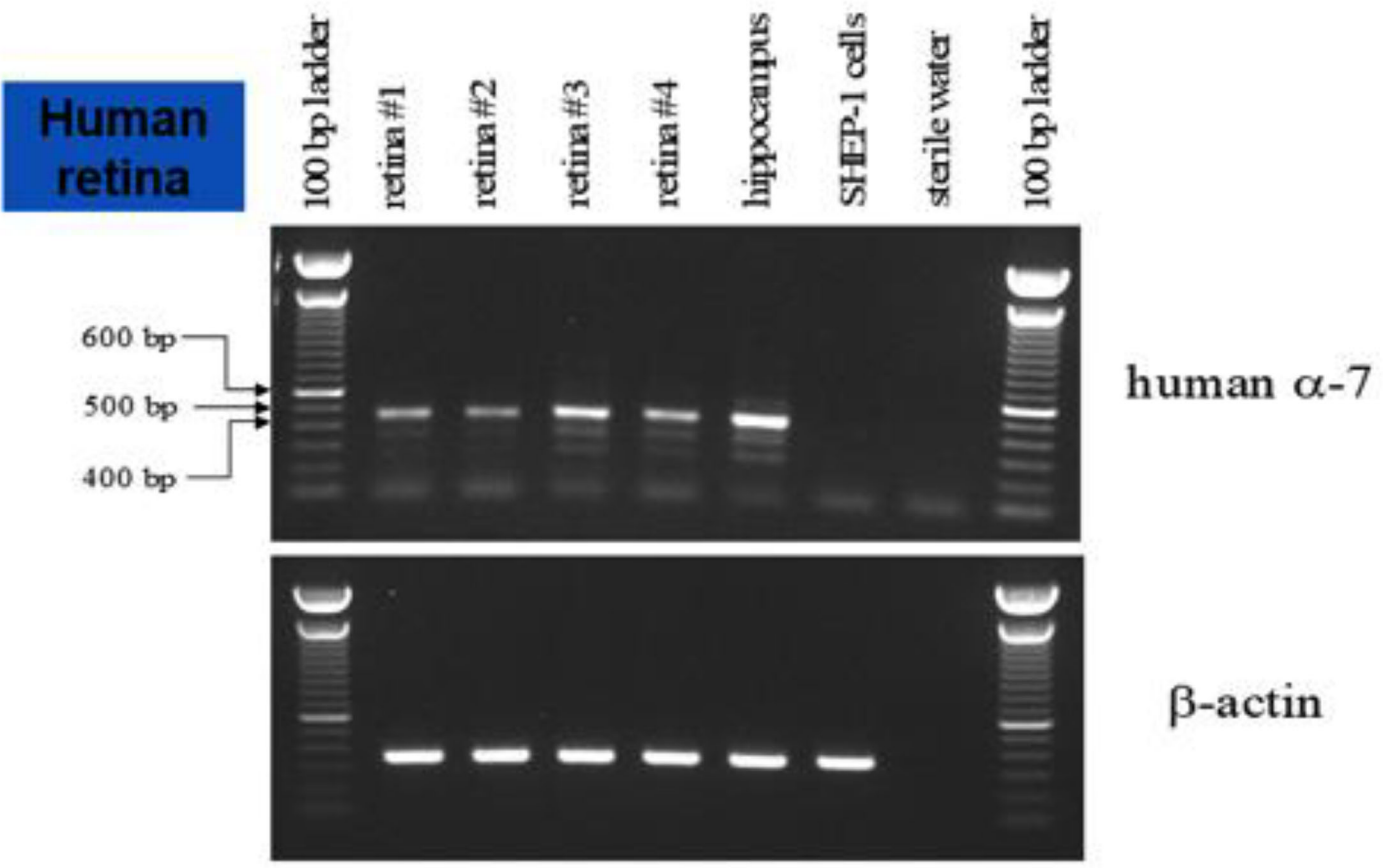
RT-PCR of the human retina. The top panel shows the results produced with the human retina, hippocampus (positive control), SHEP-1 cells (negative control), and sterile water (negative control) probed for human α7 nAChR. The bottom panel is the control experiment for β-actin with the same samples. These results support the localization of the α7 nAChR message to the human retina.

Additional RT-PCR experiments were conducted to confirm the message of α7 nAChRs in the human retina. The top portion of **Figure 5** shows the results from the human retina, hippocampus (positive control), SHEP-1 cells (negative control), and sterile water (negative control) probed for human α7. The bottom of **Figure 5** is the control experiment for β-actin with the same samples. These preliminary results support the localization of the α7 nAChR message in the human retina.

**Figure 6:**
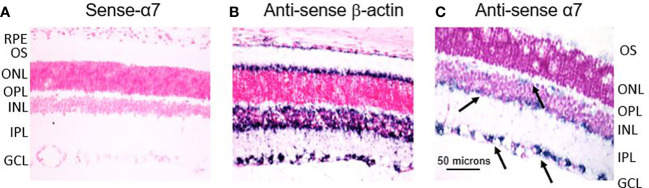
*In situ* hybridization experiments indicate the location of the α7 nAChR to the RGL of the retina. **(A)** is the sense-α7 probe serving as the negative control. The middle image **(B)** is the antisense β-actin probe serving as a positive control. The image on the right **(C)** is the antisense α7 probe showing punctate labeling throughout the retina. There is strong labeling through the GCL (lower two black arrows). There is also labeling of cells at the border of the INL and IPL, suggestive of the conventional location of cholinergic starburst amacrine cells (middle black arrow). There is also diffuse labeling at the distal border of the INL, suggestive of α7 receptors at the synapse of photoreceptors and bipolar cells (top black arrow).

ISH experiments were also conducted to confirm the location of the α7 receptor to RGCs in the retina. In **Figure 6**, the left image (A) is the α7 sense probe serving as the negative control. The middle image (B) is the β-actin antisense probe serving as the positive control. The right image (C) is the α7 antisense probe showing distinct labeling throughout the retina. There is strong labeling in the GCL (lower two black arrows). There is also labeling of cells at the border of the INL and IPL, suggestive of the conventional location of cholinergic starburst amacrine cells (middle black arrow). There is also diffuse labeling at the distal border of the INL suggestive of α7 receptors at the synapse of photoreceptors and bipolar cells (top black arrow), as others have also reported (71). However, this may also indicate horizontal cells. These results support the RT-PCR results indicating the location of the α7 nAChR to the GCL and possibly amacrine and bipolar cells (68) in the mammalian retina. These results also support the localization of α7 nAChRs to the photoreceptor outer segments and retinal pigment epithelium (RPE), as reported by Webster et al. (72) and Webster et al. (73). It appears that the β-actin labeling is specific to cones, but this cannot be confirmed. The role of β-actin in the structural integrity and physiological function of the RPE and photoreceptors is an active area of research (74). Collectively, these experiments demonstrate the identification and localization of the α7 nAChR to potential therapeutic targets in the mammalian retina.

**Figure 7:**
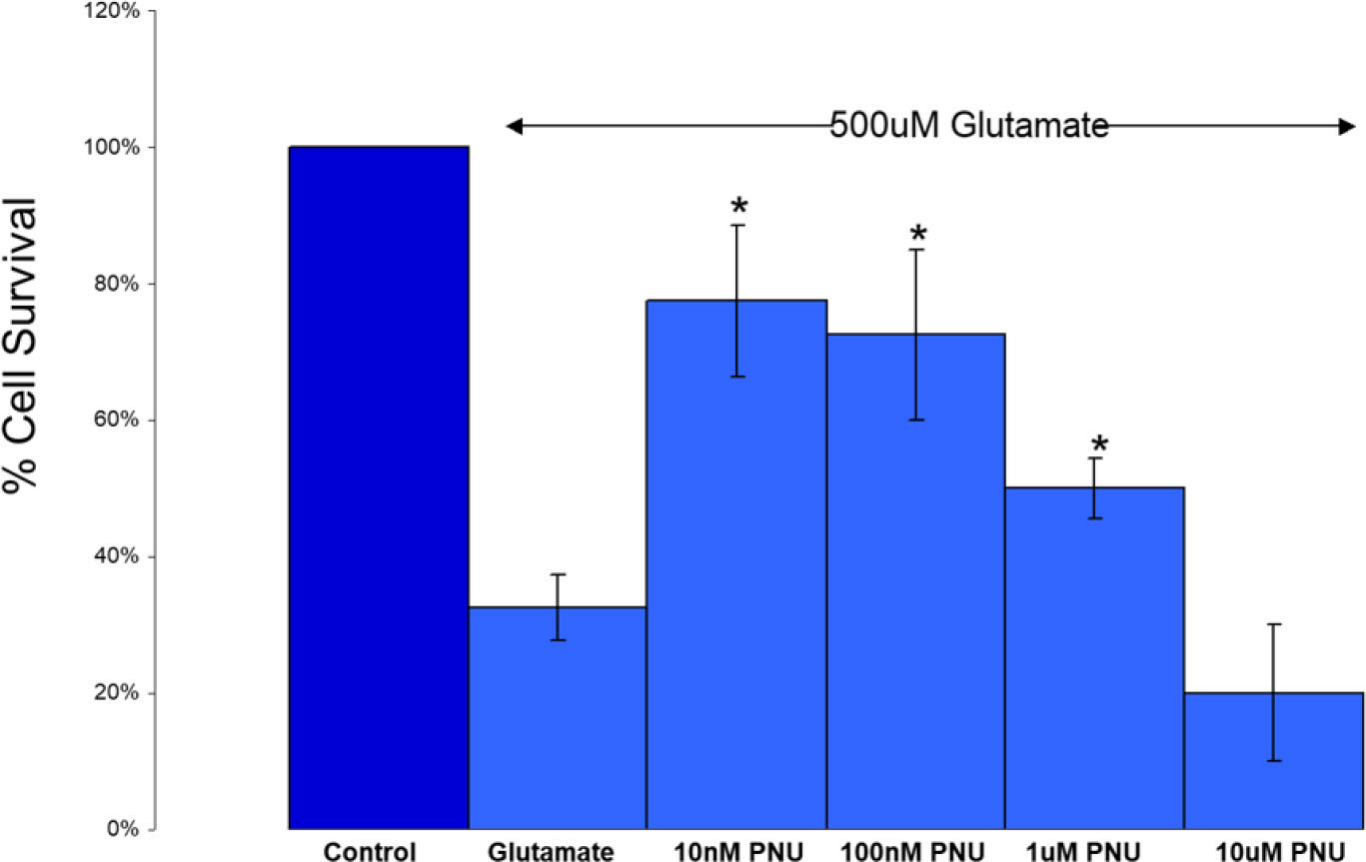
A selective α7 nAChR agonist provides protection against glutamate-induced excitotoxicity. PNU-282987 was found to be effective in the low-to-mid nanomolar concentration range (*n* = 6 for each concentration, mean ± SEM, * = significant difference from glutamate-exposed at *p* < 0.05). Additional preliminary and published experiments with nicotine also showed a drop-off in protection at higher levels (47). Additional preliminary experiments with 10 nM PNU-282987 co-applied with 10 nM α-BTX showed a reduction in the neuroprotective effects of PNU-2822987 below the level of significance. Published results using 10 nM MLA and α-BTX also inhibited the protective effects of 100 nM PNU-282987 (75). Collectively, these results support the concept that the neuroprotective mechanism of PNU-282987 is mediated through α7 nAChRs.

Validation experiments (“proof of concept”) for the utility of a selective α7 nAChR agonist in neuroprotection were conducted after the previous experiments localized the α7 nAChR target to RGCs. As mentioned in the *Introduction*, PNU-282987 is widely regarded as a highly potent and selective agonist at the α7 nAChR. Therefore, one might expect it to provide neuroprotection in a model system. One *in vitro* model that has been widely used exposes isolated RGCs [obtained through an antibody panning process, 59)] to high levels of glutamate in the presence/absence of potential neuroprotective compounds. **Figure 7** shows part of the results obtained during one such study (76). PNU-282987 was observed to be neuroprotective against glutamate-induced (500 μM) excitotoxicity. It was found to be effective in the low-to-mid nanomolar concentration range (*n* = 6 for each concentration, mean ± SEM, significant difference from glutamate-exposed at a *p*-value < 0.05). These preliminary studies were confirmed by Iwamoto et al. (75) who reported that the neuroprotective effect of 100 nM PNU-282987 against glutamate-induced toxicity was blocked with either 10 nM MLA or α-BTX with isolated rat RGCs. This supports the concept that the neuroprotective mechanism of PNU-282987 is mediated through α7 nAChRs. Follow-up studies using intravitreal injections (77) and eye-drop application (78) of PNU-282987 in the hypertonic saline injection model of glaucoma in rats support this proposed mechanism of α7 nAChR-mediated neuroprotection.

## Discussion

The preliminary target identification and validation results for the α7 nAChR in the retina can be summarized into three main findings: (1) the α7 AChR can be localized to the surface of retinal cells in the mammalian retina, (2) molecular studies support these findings by localizing the α7 nAChR to the RGL (among other locations) of mammals including humans, and (3) pharmacological cell culture studies support the concept that activation of the α7 nAChR on RGCs is neuroprotective and a potential therapeutic target for retinal diseases. Although one of the goals of this study was to establish the existence of the α7 receptor in the mammalian retina, species differences will have to be considered in further investigations of any potential therapeutic potential of a selective compound.

Interestingly, in addition to neuroprotection (i.e., preventing loss), the preliminary finding of the increased numbers of cells in the porcine retinal cell culture above control levels *via* α7 nAChR activation by PNU-282987 (79) was confirmed with isolated rat RGCs (75). This increase in RGC cell numbers was confirmed in the intact rat following 2 mM PNU-282987 eye-drop application (78). This raised the possibility that new cells were being generated with PNU-282987 in adult animals following eye-drop application. Why is neuroprotection the predominant effect with PNU-282987 following intravitreal injection and in RGC cell culture, while putative neurogenesis is observed with eye-drop application? One could postulate that RGCs receive a higher dose of PNU-282987 from the vitreous, as the compound diffuses past them first before other cells following intravitreal injections. This would lead to a predominantly neuroprotective effect on RGCs *via* activation of α7 nAChRs. Conversely, eye-drop application has a predominant effect on other cells in the retina (possibly outer retinal cells including the RPE) as the compound diffuses past them first toward the RGL, leading to proliferation. Webster et al. (72) elegantly demonstrated that this effect is mediated by α7 nAChRs on the RPE, which then direct Müller glia (MG) to dedifferentiate to produce other retinal neurons. The most recent evidence (73) indicates that PNU-282987 causes a bi-modal signaling event in which early activation primes the retina with an inflammatory response and developmental signaling cues, followed by an inhibition of gliotic mechanisms and a decrease in the immune response. This culminates with the upregulation of genes associated with specific types of retinal neuron generation. Taken together, these data provide evidence that PNU-282987 activates the retinal pigment epithelium to signal MG to produce MG-derived progenitor cells, which can differentiate into new functional neurons in adult mice. These studies not only increase our understanding of how adult mammalian retinal regeneration can occur, but also provide therapeutic promise for treating the loss of specific retinal neurons in different disease states [e.g., retinitis pigmentosa and glaucoma, (80)].

However, do these newly generated cells produce functional connections in retinal models of disease and trauma? Preliminary (81) and recently published results suggest they do (82). In the hypertonic saline injection model of glaucoma, new cells have been shown to proliferate when the injection procedure to elevate intraocular pressure is followed by PNU-282987 eye drops. Four weeks after daily eye-drop application the changes due to the injection procedure largely restore retinal morphology and ERG function to preinjection levels. In addition, Spitsbergen et al. (82) published reports showing that eye-drop application of PNU-28287 following a simulated “blast-damage” to the eye also restored retinal morphology and ERG response to a near pre-blast status. These preliminary and published results need to be confirmed and expanded into possible therapeutic avenues for treating vision loss due to disease and trauma.

The retinal “cholinergic tone” theory is supported by the observation that starburst amacrine cells are the first to die following the hypertonic saline procedure (31). One could propose that “replacement therapy” with a selective α7 nAChR agonist to substitute for the decrease in ACh release is effective in restoring “cholinergic tone” and providing neuroprotection for RGCs. However, the sequential process underlying the proliferative effect following α7 nAChR activation on the RPE and the subsequent MG dedifferentiation does not appear to be as straightforward. If the cholinergic (starburst) amacrine cells are the only cells in the adult mammalian retina to produce ACh, it would follow that RGCs should be targets and possess receptors. But why would the RPE located distally from the IPL (inner plexiform layer; i.e., site of ACh release) of the retina also possess functional α7 nAChRs?

One theory could be that during development, the nAChRs on the RPE are involved in the development of the eye, for example by regulating eye growth (83). This is based, in part, on the observation that a compound (chlorisondamine), which accumulates in neurons with nicotinic receptors, induced RPE layer degeneration. Maneu et al. (84) later provided evidence that α7 nAChRs are present on the RPE in the adult retina based on RT-PCR, immunohistochemistry (IHC), and Fura-2 imaging studies. These results led the authors to suggest that nAChRs could have a significant role in RPE physiology, which may not be related to a more traditional role in nerve transmission. It could more likely be related to the non-neuronal cholinergic system in the eye. Furthermore, in a model of long-term hypoxia, they reported that expression of α7 nAChRs was downregulated. This led them to propose that α7 nAChRs in the RPE could be involved in cell protection mechanisms (84). Regardless of the specifics of the origin of α7 nAChRs on the RPE, they do exist and after activation can induce MGs to generate new retinal cells that offer a therapeutic potential above and beyond the neuroprotection observed with the activation of α7 nAChRs on RGCs. This could lead to exciting possibilities in treating conditions that normally result in irreversible loss of vision.

## Data availability statement

The original contributions presented in the study are included in the article. Further inquiries can be directed to the corresponding author.

## Ethics statement

The animal study was reviewed and approved by the Institutional Animal Care and Use Committee at Pharmacia & Upjohn.

## Author contributions

The author confirms that they are the sole contributor to this work and has approved it for publication.
